# Recent Advances on Natural Aryl-*C*-glycoside Scaffolds: Structure, Bioactivities, and Synthesis—A Comprehensive Review

**DOI:** 10.3390/molecules27217439

**Published:** 2022-11-01

**Authors:** Chen-Fu Liu

**Affiliations:** School of Pharmaceutical Sciences, Gannan Medical University, Ganzhou 341000, China; chenfu@gmu.edu.cn

**Keywords:** aryl-*C*-glycoside, novel structures, bioactivity, synthesis

## Abstract

Aryl-*C*-glycosides, of both synthetic and natural origin, are of great significance in medicinal chemistry owing to their unique structures and stability towards enzymatic and chemical hydrolysis as compared to *O*-glycosides. They are well-known antibiotics and potent enzyme inhibitors and possess a wide range of biological activities such as anticancer, antioxidant, antiviral, hypoglycemic effects, and so on. Currently, a number of aryl-*C*-glycoside drugs are on sale for the treatment of diabetes and related complications. This review summarizes the findings on aryl-*C*-glycoside scaffolds over the past 20 years, concerning new structures (over 200 molecules), their bioactivities—including anticancer, anti-inflammatory, antioxidant, antivirus, glycation inhibitory activities and other pharmacological effects—as well as their synthesis.

## 1. Introduction

Aryl-*C*-glycosides (ACGs), natural secondary metabolites, are glycosides in which the anomeric center is covalently linked to the carbon atom of arenes or heterocycles (C_sp3_-C_sp2_) [1]. They tend to have high oral bioavailability and reach high plasma levels without needing to be converted to a prodrug. The stable linkage between the sugar and the arene or heterocycle moieties is resistant to enzymatic hydrolysis, allowing these compounds to interfere with DNA and RNA synthases more efficiently [2]. Both natural and synthetic aryl-*C*-glycosides are of marked pharmaceutical interest, and many of them have proven to be efficient antibiotics, antitumor agents, and antidiabetics. Therefore, aryl-*C*-glycosides have received considerable attention [3].

Beginning with the structural elucidation of the first aryl-*C*-glycoside in 1970 [4], the study of aryl-*C*-glycosides has continued to flourish through the constant isolation/characterization of new aryl-*C*-glycoside natural products, the characterization of the corresponding biosynthetic pathways/enzymes, and the development of aryl-*C*-glycoside synthetic methods. As their name suggests, the core structure of aryl-*C*-glycosides is often abundantly decorated with substituents such as aromatic acids (e.g., gallic acid, caffeic acid, vanillic acid, benzoic acid, and ferulic acid) and various saccharides (e.g., L-rhamnose, D-xylose, D-glucose, D-galactose, and L-arabinose) through ester or glycosidic linkages, respectively. The outstanding activity of aryl-*C*-glycosides against diverse diseases proves their importance in medicinal chemistry research. Several reviews on aryl *C*-glycosides regarding their isolation and purification, structure elucidation, synthesis and biosynthesis, and pharmacological activities have been published [5,6,7]. Recently, interest in aryl-*C*-glycoside has been growing, as shown by a significantly increasing volume of literature describing novel structures, diverse bioactivities, general synthesis, and evident roles of these molecules in the prevention and treatment of various human diseases [8]. Such rich information prompted me to review papers on novel aryl-*C*-glycoside structures, pharmacological activities, and chemical synthesis published in the last two decades. This review will highlight the new structures (over 200 new aryl-*C*-glycoside molecules), bioactivities, and synthetic approaches to the preparation of aryl-*C*-glycosides, which were published in prestigious journals such as Journal of the American Chemical Society, Angewandte Chemie International Edition, Journal of Natural Products, Organic Letters, Phytochemistry and in related peer-reviewed natural product research journals, from 2002 to 2022. A summary of the structures, bioactivities, and origins of recently discovered aryl-*C*-glycoside molecules is provided in Table 1.

## 2. Structures

### 2.1. Flavonoid C-Glycosides

Apigenin and luteolin represent the major parent nuclei of flavonoid *C*-glycosides; the most common glycan moieties linked to *C*-glycosides are D-glucose, D-galactose, D-xylose, D-mannose, D-ribose, L-fucose, L-arabinose, and L-rhamnose.

#### 2.1.1. Flavonoid *C*-Glucosides

To the best of my knowledge, Flavonoid *C*-*α*-D-glucopyranosides, Flavonoid *C*-*α*-L-glucopyranosides, and Flavonoid *C*-*β*-L-glucopyranosides have not been isolated and identified from natural source. Flavonoid *C*-*β*-D-glucopyranosides are the largest group of isolated aryl-*C*-glycosides. Since 2002, more than 80 new compounds have been isolated and identified. It was found that the D-glucose unit is directly attached to the flavonoid or flavonoid derivatives in *β* configuration. Compared with the known aryl-*C*-glycosides previously reported, some of the new ones differ in their core structure, while others differ in the number and/or position of their substituents. The sites of glycosylation in the flavonoids are usually C6 and/or C8. Very few examples are known of *C*-glycosylation occurring at position C4′.

**Figure 1:**
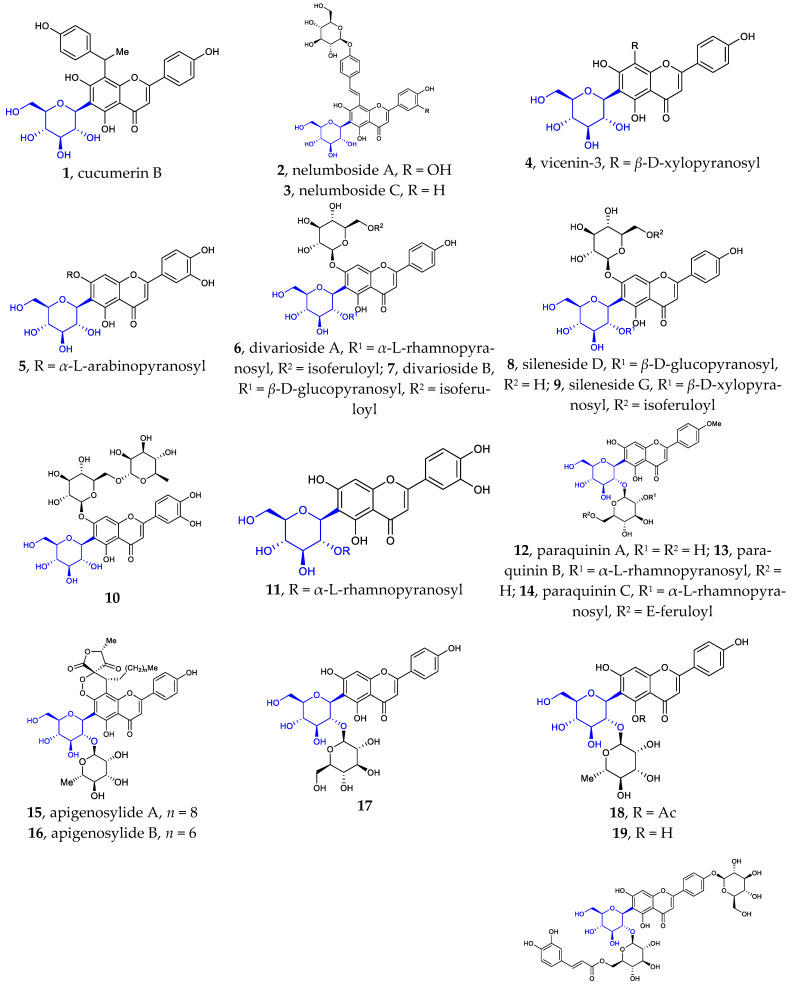

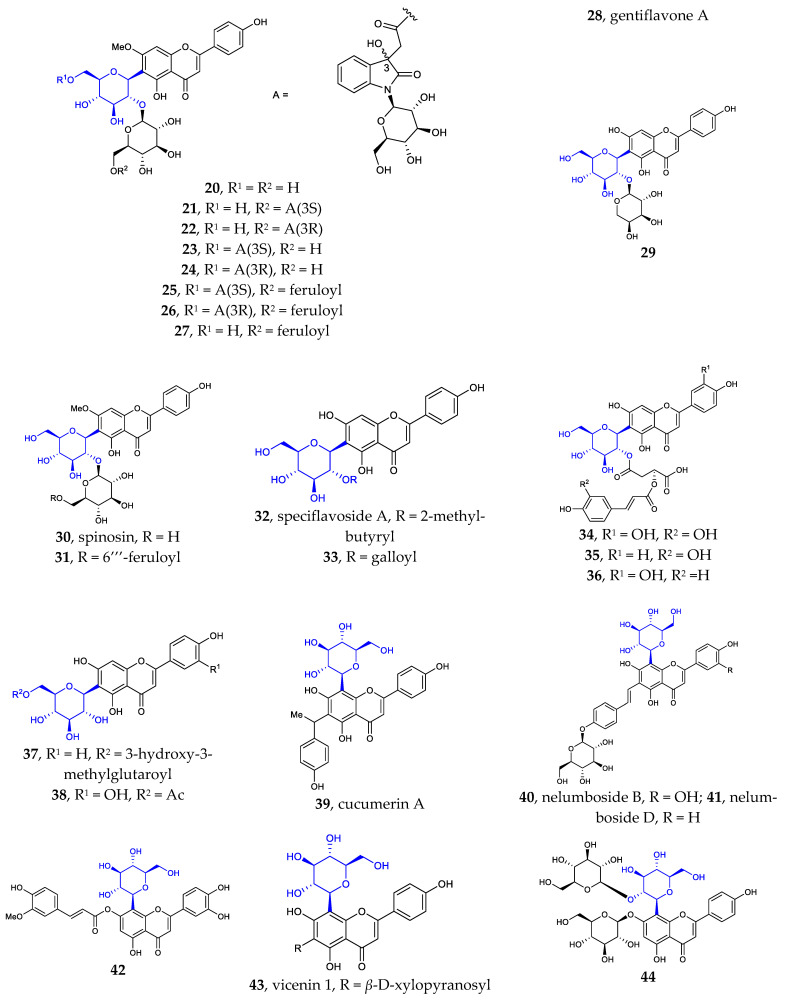

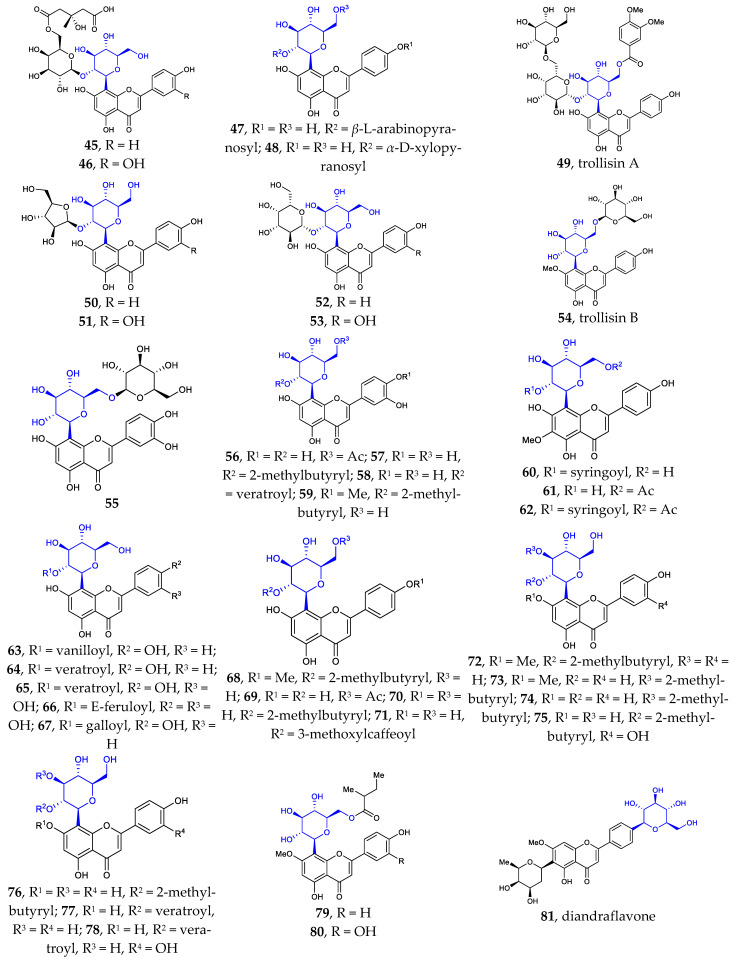
Chemical structures of flavonoid 6- or 8-*C*-*β*-D-glucopyranosides (**1**-**80**) and flavonoid 4′-*C*-*β*-D-glucopyranoside (**81**).

Figure 1 illustrates the new aryl-*C*-glycosides with varied core structures or special substituents. These structures differ in the aglycon and or glucoside portions. The core structures are often abundantly decorated with substituents such as aromatic acids (e.g., benzoic acid, 2-methyl butyric acid, gallic acid, veratric acid, sinapic acid, ferulic acid, and acetic acid) and various saccharides (e.g., L-rhamnose, D-xylose, D-glucose, D-galactose, and L-arabinose) through ester or glycosidic linkages, respectively. These isolated structures are often associated with other known compounds, such as flavonoids, lignan, sesquiterpene, steroid, alkaloids, *O*-glycosides, etc.

Besides the well-known flavonoid *C*-glycosides vitexin, isovitexin, orientin, isoorientin, schaftoside, isoshaftoside, neoshaftoside, and their *O*-glycosylated and or *O*-acylated derivatives, more and more flavonoid *C*-glycosides were disclosed. These 6- or 8-*C*-*β*-D-glucopyranosides can be divided into five groups: (1) mono-*C*-glycosylated flavonoids **1**–**3** [12,37], **39**–**42** [12,13,37]; (2) 6, 8-di-*C*-glycosylated flavonoids **4** [38], **43** [39]; (3) flavonoid 6- or 8-*C*-*β*-D-glucopyranoside-7-*O*-*β*-D-glucopyranosides **5**–**10** [40,41,42,43], **44** [44]; (4) 2″- or 6″-*O*-glycosylated flavonoid 6- or 8-*C*-*β*-D-glucopyranosides **11**–**31** [9,38,45,46,47,48,49,50], **45**–**55** [14,51,52,53,54,55,56,57]; (5) 2″- or 6″-*O*-acylated flavonoid 6- or 8-*C*-*β*-D-glucopyranosides **32**–**38** [10,11,55,56], **56**–**80** [57,58,59,60,61]. Some of the above compounds are glycosylated with *β*-D-glucose, a disaccharide consisting of *β*-D-glucose glycosylated with a monosaccharide at position C2 or C6, or *β*-D-glucose acylated at C2 or C6. Such glycosylation with various sugars and/or acylation with various acids is a common strategy used by nature to introduce structural diversity and different biological activities in natural products.

It is noteworthy that flavonoid di-*C*-glycoside **81** features the *β*-D-glucopyranosyl and *β*-D-oliopyranosyl groups attached to C-4′ and C-6, respectively [16]. This kind of compound is very rare in nature.

#### 2.1.2. Flavonoid *C*-Galactosides, *C*-Arabinosides, *C*-Xylosides, *C*-Mannosides, *C*-Fucosides, *C*-Boivinosides, and *C*-Riboside

Compared with flavonoid *C*-glucosides, flavonoid *C*-galactosides are less common in nature. Compounds **82**–**94** have diversified structures which can be classified into two groups: (1) flavonoid 6-*C*-*β*-D-galactopyranosides, **82**–**85** [62,63,64]; (2) flavonoid 8-*C*-*β*-D-galactopyranosides, **86**–**94** [64,65,66]. To date, no flavonoids galactosylated at other sites than C6 and C8 have been discovered. The hydroxyl groups of D-galactose or other sugars are often acylated or glycosylated, which leads to multiple complex structures with unique functions.

**Figure 2:**
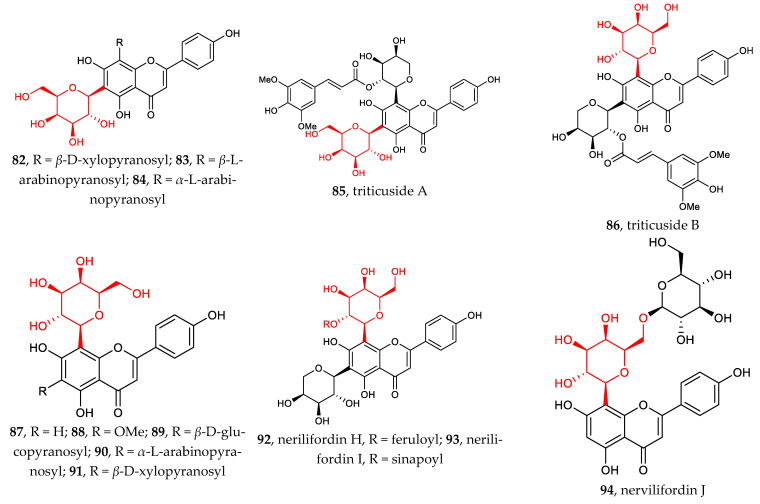

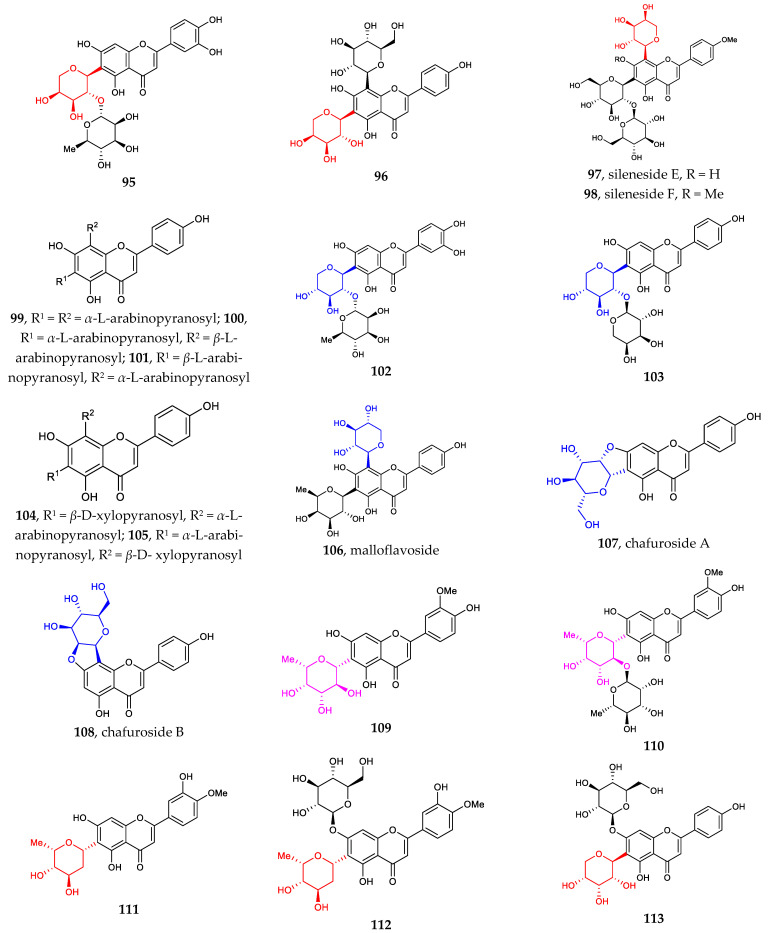
Chemical structures of flavonoid *C*-galactosides (**82**–**94**), *C*-arabinosides (**95**–**101**), *C*-xylosides (**102**–**106**), *C*-mannosides (**107**–**108**), *C*-fucosides (**109**–**110**), *C*-boivinosides (**111**–**112**), and *C*-riboside (**113**).

Figure 2 illustrates the recently discovered structures of flavonoid *C*-arabinosides **95**–**101** [67,68,69]. Di-*C*-glycosylation in flavonoids **96**–**101** usually takes place at positions C6 and C8. Both *C*-*α*-L-arabinosides and *C*-*β*-L-arabinosides are associated with *β*-D-glucose, *β*-D-galactose, and *β*-D-Xylose, with glycosylation sites shifting between C6 and C8.

The sites of *C*-glycosylation of flavonoid *C*-xylosides are usually C6 and or C8. Compounds **102**–**106** can be divided into two groups: (1) flavonoid mono-*C*-xylopyranosides **102**–**103** and (2) di-*C*-glycosylflavonoids **4**, **43**, **82**, **91**, **104**–**106** [38,39,62,69,70]. All discovered flavonoid *C*-xylosides are in *β* configuration.

Other flavonoid *C*-glycosides, including flavonoid *C*-mannosides **107**–**108**, flavonoid *C*-fucosides **109**–**110**, flavonoid *C*-boivinosides **111**–**112**, and flavonoid *C*-riboside **113**, are rarely found in the nature [71]. The site of *C*-glycosylation is normally C6.

### 2.2. Other Flavonoid C-Glycosides

**Figure 3:**
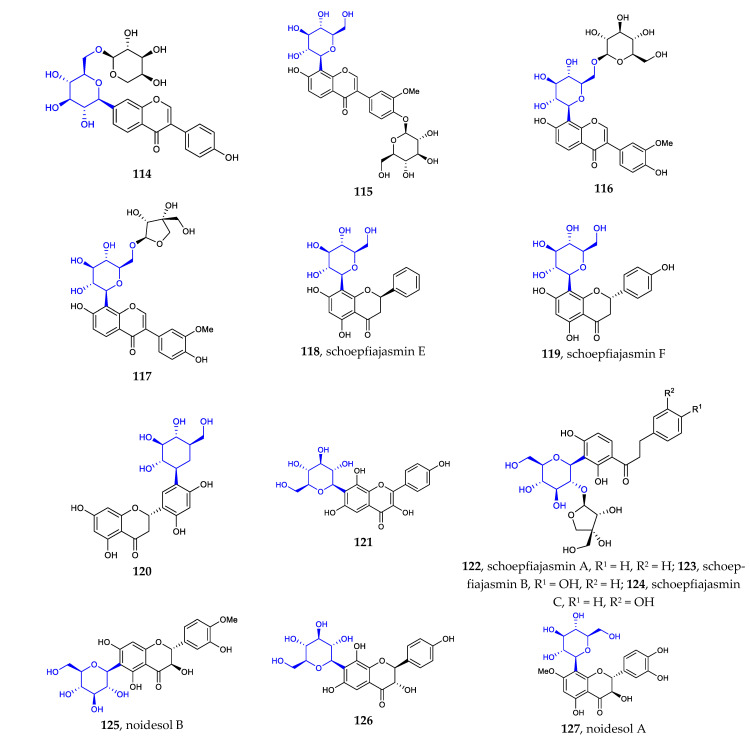
Chemical structures of isoflavone *C*-*β*-D-glycosides (**114**–**117**), flavanone *C*-glycosides (**118**–**120**), flavonol *C*-glycoside **121**, dihydrochalcone *C*-glycosides (**122**–**124**), and flavanonol *C*-glycosides (**125**–**127**).

Other flavonoid *C*-glycosides include isoflavone [72,73], flavanone [74,75], dihydrochalcone [74], flavanonol [76,77], and flavonol *C*-glycosides [76]. The newly isolated structures are illustrated in Figure 3. Among them, Compound **120** possesses a unique scaffold featuring a *C*-*β*-D-glucose core linked to the flavanone at position C5′. This type of aryl-*C*-glycoside is a rare example of a natural aryl *C*-glycoside. It is very clear that all compounds are *C*-*β*-D-glucopyranosides (**114**–**127**), some of which are glycosylated with a monosaccharide at position C2 or C6 (Figure 3).

### 2.3. Xanthone C-Glycosides

**Figure 4:**
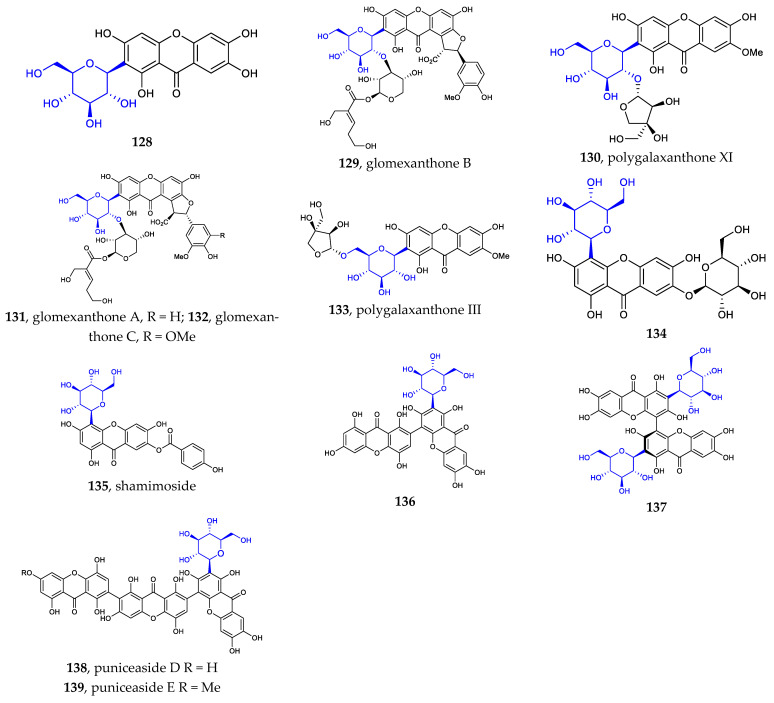
Chemical structures (**128**–**139**) of xanthone *C*-glycosides.

The sites where the sugar is linked to the xanthone are usually C2 (**128**–**133**, **136**–**139**) and C4 (**134**–**135**) [21,22,23,78,79]. It seems that only *C*-*β*-D-glucosides have been isolated from nature. This kind of aryl *C*-glycosides include mono-xanthone *C*-glycosides, di-xanthone *C*-glycosides, and tri-xanthone *C*-glycosides (Figure 4).

### 2.4. Phenyl C-Glycosides

**Figure 5:**
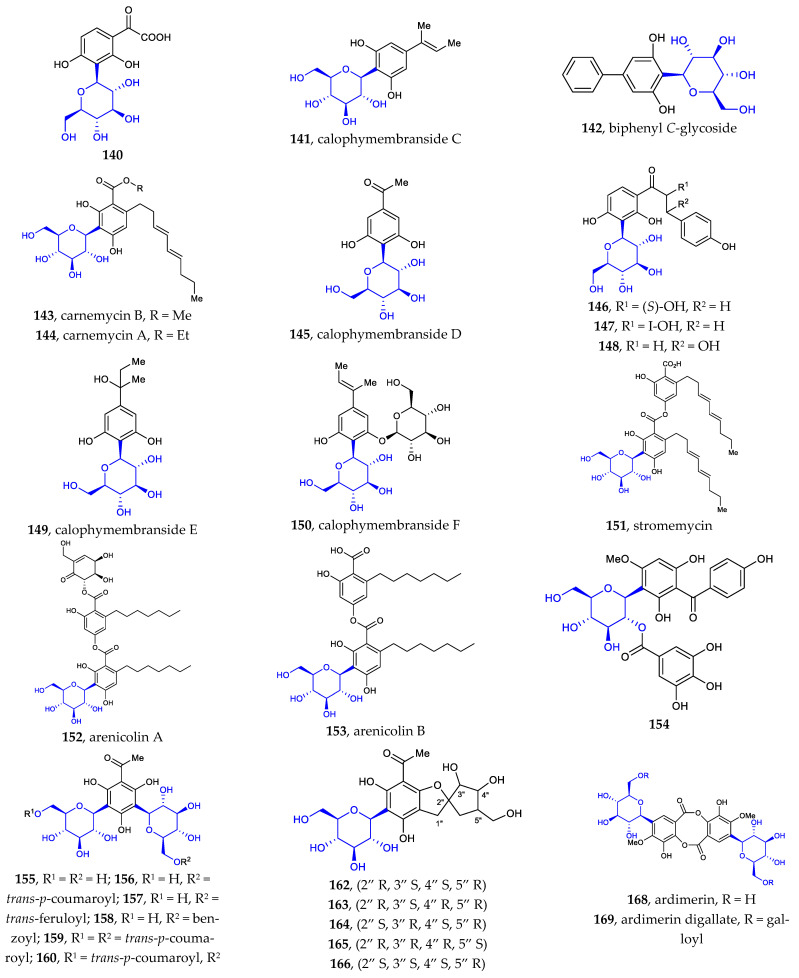

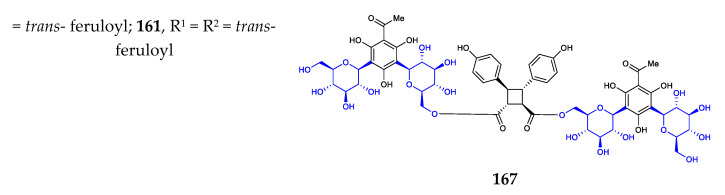
Chemical structures (**140**–**169**) of phenyl *C*-glycosides.

Phenyl *C*-glycosides were considered the simplest aryl-*C*-glycosides. The disclosed structures **140**–**169** feature *C*-*β*-D-glucopyranosides, with variations in the substitution on the benzene ring (Figure 5) [24,25,26,27,80,81,82,83,84]. The site of *C*-glycosylation is usually the ortho position of the phenolic hydroxyl group. The substitution groups of benzene include acyl, alkenyl, hydroxyl, alkyl, and alkoxyl moieties, providing aryl ketone, aryl vinyl, phenol, alkylbenzene, and aryl ether, respectively. The 2′-hydroxylation and 6′-hydroxylation of D-glucose are often accompanied by esterification by gallic acid (compounds **154**, **169**), *trans*-*p*-coumaric acid (compounds **156**, **159**–**160**), ferulic acid (compounds **157**, **160**–**161**), and benzoic acid (compound **158**).

### 2.5. Heteroaryl C-Glycosides

**Figure 6:**
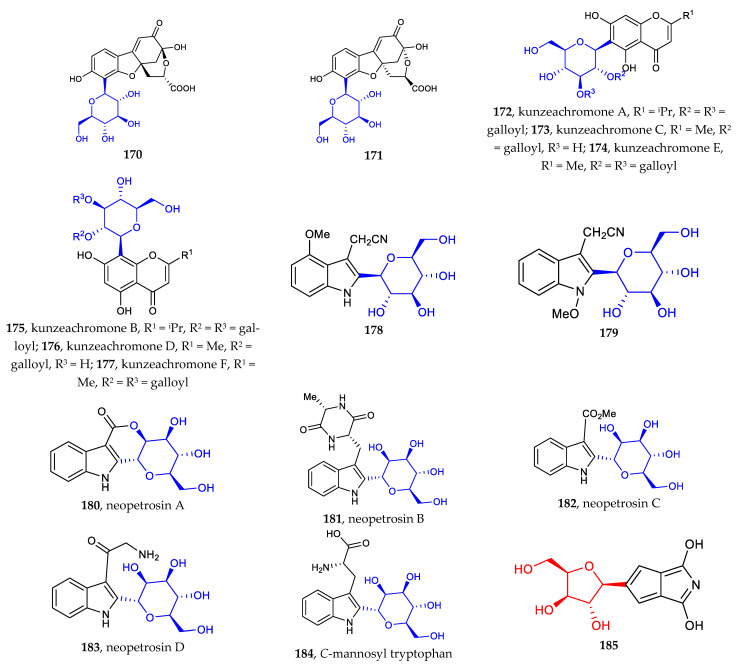

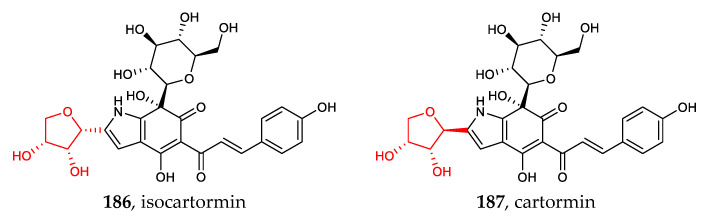
Chemical structures (**170**–**187**) of heteroaryl *C*-glycosides.

The recently discovered heteroaryl *C*-glycosides include dihydrobenzofuran *C*-glycosides (**170**–**171**), chromone *C*-glycosides (**172**–**177**), indole *C*-glycosides (**178**–**184**), and other heteroaryl *C*-glycosides (**185**–**187**) [28,29,85,86]. Except for the common *C*-*β*-glucosides, *C*-*α*-D-mannopyranosides and those containing erythrose are rare examples of aryl-*C*-glycosides (Figure 6).

### 2.6. Other Aryl-C-Glycosides

**Figure 7:**
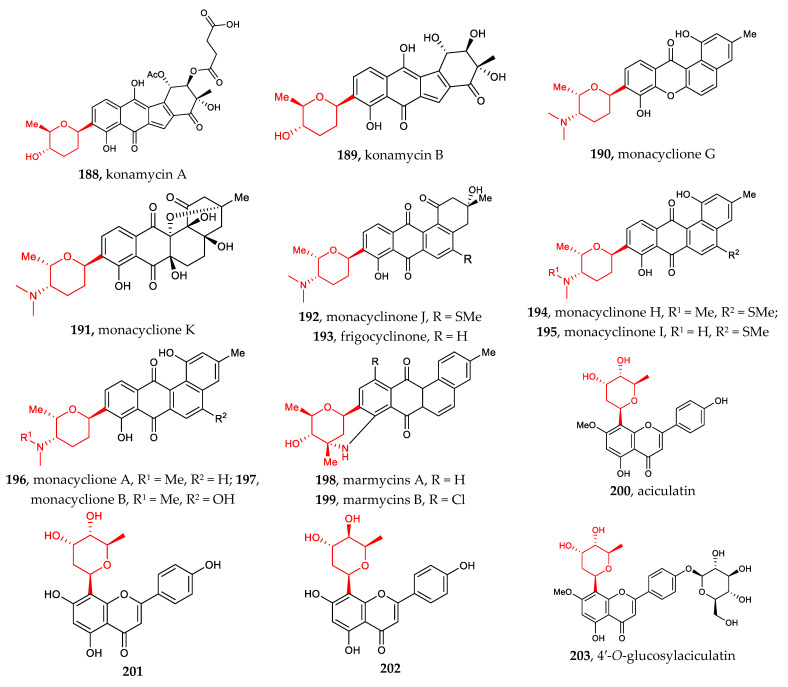

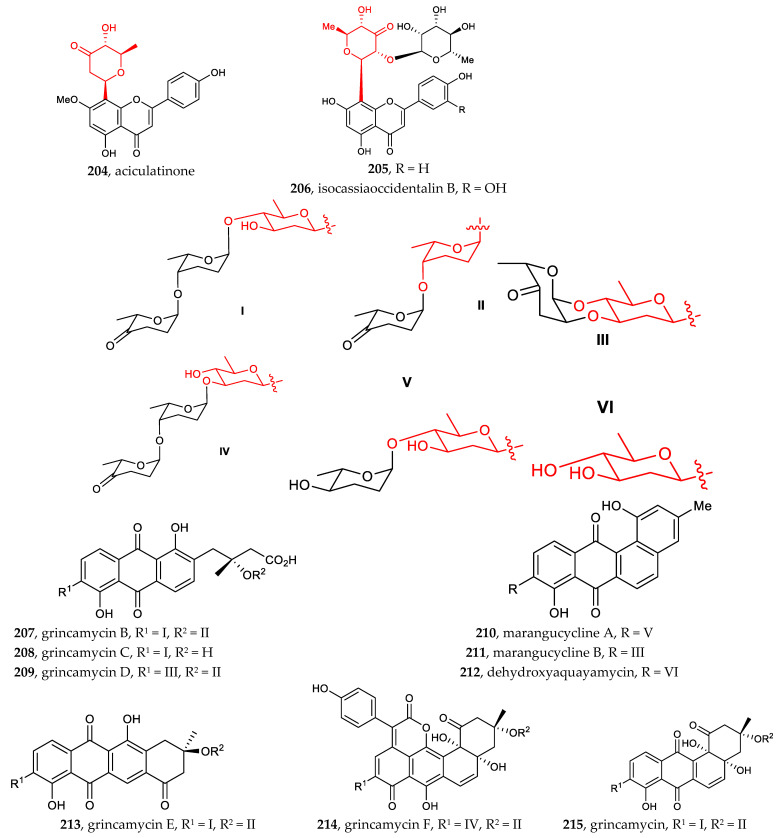
Chemical structures (**188**–**215**) of aryl *C*-glycosides.

Other aryl-*C*-glycosides include aryl-*C*-glycosides of amino sugars and other rare sugars [30,31,32,33,34,35,36]. Compounds **190**–**197** (4-aminosugar) and compound **198**–**199** (3-aminosugar), which are 2-deoxylaminoglycoside antibiotics, resemble the well-known pluramycins. Among them, monacyclione G (**190**) possesses a unique scaffold featuring a xanthone core linked to the aminodeoxysugar ossamine, and monacycliones H−J (**194**–**195**, **192**) are rare examples of natural angucyclines with an S-methyl group (Figure 7).

## 3. Pharmacological Activity

Numerous researchers have investigated the pharmacological activities of various aryl-*C*-glycosides. Table 1 summarizes the pharmacological features of recently discovered aryl-*C*-glycoside molecules. They include, but are not limited to, anticancer, anti-inflammatory, antioxidant, antiviral activities, glycation inhibitory activity, other pharmacological activities such as neuroprotective effects, hepatoprotective activity, and antipyretic activity. These pharmacological activities are summarized below.

### 3.1. Anticancer Activity

The indole *C*-glucopyranoside **178** exhibited significant cytotoxic activity against human myeloid leukemia cells HL-60 and human liver cancer cells HepG2, with IC50 of 1.3 ± 0.1 and 2.1 ± 0.3 μM, respectively. The indole *C*-glucopyranoside **179** showed potential cytotoxic activity against HL-60 and human myeloid leukemia Mata cells, with IC_50_ of 5.1 ± 0.4 and 12.1 ± 0.8 μM, respectively [85]. Monacycliones I (**195**) and J (**192**), isolated from the marine-derived *Streptomyces* sp. HDN15129, showed cytotoxic activity against multiple human cancer cell lines, with IC_50_ values ranging from 3.5 to 10 μM [31]. Marmycin A (**198**), isolated from the culture broth of a marine sediment-derived actinomycete related to the genus *Streptomyces*, displayed significant cytotoxicity against several cancer cell lines, some at nanomolar concentrations, while marmycin B (**199**) was less potent. For marmycin A (**198**), tumor cell cytotoxicity appeared to coincide with a modest induction of apoptosis and the arrest in the G1 phase of the cell cycle [32]. Li et al. reported that compounds **34**–**36**, isolated from the small flowering aquatic plant *Lemna japonica*, exhibited weak cytotoxicity against HepG-2, SW-620, and A-549 cell lines, with IC_50_ values between 42.5 and 19.2 μg/mL [11]. Isoorientin, recently isolated from leaf and root methanolic extracts of *Petrorhagia Velutina*, a Mediterranean herbaceous plant, significantly reduced the proliferation of HepG2 cells, as determined by the complete conversion of a tetrazolium probe into formazan after 48 h of exposure [67]. Aciculatin **200**, **201**, **202**, **204**, isolated from an ethanolic extract of *Chrysopogon aciculatis*, showed differential potency on different cancer cell lines. Noticeably, aciculatin and **201** indicated specificity of cytotoxicity in MCF-7 and CEM cell lines [33]. Grincamycins B-E (**207**–**209**, **213**), isolated from *Streptomyces lusitanus* SCSIO LR32, exhibited in vitro cytotoxicity against the human cancer cell lines HepG2, SW-1990, HeLa, NCI-H460, and MCF-7 and the mouse melanoma cell line B16, with IC50 values ranging from 1.1 to 31 μM [35]. Marangucycline B (**211**), isolated from the deep-sea-derived *Streptomyces* sp. SCSIO 11594, displayed in vitro cytotoxicity against four cancer cell lines, i.e., A594, CNE2, HepG2, and MCF-7, superior to that obtained with cisplatin, used as a positive control. Notably, marangucycline B bearing a keto-sugar displayed significant cytotoxicity against various cancer cell lines, with IC_50_ values ranging from 0.24 to 0.56 μM. An IC_50_ value of 3.67 μM was found when using the non-cancerous hepatic cell line HL7702, demonstrating the cancer cell selectivity of marangucycline B [36].

### 3.2. Anti-Inflammatory Activity

Compound **68**, isolated from the rhizomes of *Cyperus rotundus*, showed moderate inhibitory activity against MRB, with an IC_50_ value of approximately 56.03 µM [15]. Nervilifordin J (**94**), isolated from a 60% EtOH extract of the aerial parts of *Nervilia fordii*, showed interesting inhibitory effects on nitric oxide production in lipopolysaccharide-activated RAW264.7 macrophages, with EC_50_ values of 14.80 µM [17]. Vicenin-2 was isolated and identified from an ethanol extract of the aerial parts of *Urtica circularis*. This crude extract was found to possess significant anti-inflammatory activity in a carrageenan-induced rat hind paw edema model (41.5% inhibition at a dose of 300 mg/kg). In cultured murine macrophages, this compound modified LPS-induced total nitrite and TNF-α production, in addition to promoting the LPS-induced translocation of nuclear factor NF-κB [87].

### 3.3. Antioxidant Activity

Shamimoside (**135**), isolated from a methanolic extract of the leaves of Bombax ceiba, showed antioxidant potential (IC_50_ = 150 µg/mL) [22]. Nelumboside B (**40**) exhibited strong scavenging activity (SC_50_ = 14.12 µM, ABTS assay), compared with the positive control L-ascorbic acid (SC_50_ = 26.15 µM, ABTS assay) [12]. Isocassiaoccidentalin B (**206**), isolated from whole *Cassia nomame* (SIEBER) HONDA plants, showed significant free-radical scavenging activity [34]. Compound **42**, isolated from *Gentiana piasezkii*, showed significant free-radical scavenging activity (IC_20_ = 5.20 ± 0.10 µM) in the DPPH assay [13]. Compound **113**, isolated from the methanolic extracts of *Dtps*. Tinny Ribbon × *Dtps*. Plum Rose (*Phalaenopsis hybrids*), exhibited moderate α, α-diphenyl-β-picrylhydrazyl free-radical scavenging activity, with half-maximal inhibitory concentration (IC_50_) values of 27.3 µM, compared to the reference compound vitamin E (IC_50_ 12.5 µM) [71].

### 3.4. Antiviral Activity

Speciflavoside A (**32**), isolated from a 70% methanolic extract of *Lilium speciosum* var. *gloriosoides* Baker, showed potent antiviral activity against RSV, with an IC_50_ value of 2.9 μg/mL, comparable to that of ribavirin, an approved drug for the treatment of RSV infections in humans [10].

### 3.5. Glycation Inhibitory Activity

In 2003, Okuyama T. et al. reported that chrysoeriol 6-*C*-*β*-fucopyranoside **109**, isolated from the style of *Zea mays* L. showed an inhibitory effect on glycation, with a percent inhibition value greater than that of aminoguanidine, a known glycation inhibitor [19]. Compounds **130**–**131,** which contains the rare sugar boivinose, exhibited a glycation inhibitory activity similar to that of aminoguanidine [20].

### 3.6. Other Pharmacological Effects

Other pharmacological effects include neuroprotective effects, hepatoprotective activity, HIV inhibitory activity, antipyretic activity, transcriptional inhibitory activity of RXRα, cytotoxicity activity, and other activities.

Glomexanthones A–C (**131**, **129**, **132**), isolated from an ethanol extract of *Polygala glomerata*, showed moderate neuroprotective effects on L-glutamic acid-induced cellular damage in human neuroblastoma SK-N-SH cells [21]. Compound **136**, isolated from the entire plant of *Swertia punicea*, exhibited potent neuroprotective activity against H_2_O_2_-induced PC12 cell damage [23]. Neopetrosins A, B, D (**180**–**181**, **183**) were isolated from the marine sponge *Neopetrosia chaliniformis* collected off Xisha Island in the South China Sea. They exhibited in vivo hepatoprotective activity in a zebrafish model at a concentration of 20 μM [29]. Ardimerin digallate (**169**) was isolated from the whole plant of *Ardisia japonica* and was shown to inhibit HIV-1 and HIV-2 RNase H in vitro, with IC_50_ values of 1.5 and 1.1 µM, respectively [27]. The compound 3,5-di-*C*-*β*-D-glucopyranosyl phloroacetophenone (**155**), isolated from the edible leaves of *Melicope pteleifolia*, was found to be responsible for the antipyretic activity of *M. pteleifolia* based on in vivo experiments [26]. Calophymembranside C (**141**), isolated from the stems of *Calophyllum membranaceum*, showed transcriptional inhibitory activity towards RXRα, with 50% inhibitory concentration (IC_50_) values of 29.95 ± 1.08 [24]. Arenicolin A (**152**), isolated from *Penicillium arenicola*, exhibited cytotoxicity toward mammalian cell lines, including colorectal carcinoma (HCT-116), neuroblastoma (IMR-32), and ductal carcinoma (BT-474) cells, with IC_50_ values of 7.3, 6.0, and 9.7 μM, respectively [73]. Apigenosylide B (**16**), isolated from an EtOH extract of the leaves of *Machilus japonica* var. *kusanoi*, possesses moderate inhibitory activity against α-glucosidase [9]. Compounds **45**–**46** inhibited complement activation in the classic pathway in vitro, with IC_50_ values ranging from 0.88 to 4.02 mM. This may suggest the application of the herb for the treatment of acute respiratory distress syndrome, etc. [14]. Diandraflavone (**81**), isolated from *Drymaria diandra*, showed significantly selective inhibition on superoxide anion generation from human neutrophils stimulated by fMLP/CB, with an IC_50_ value of 10.0 µg/mL [16].

## 4. Synthesis

In nature, most *C*-glycosides are derived from plants. Aryl *C*-glycosides are biosynthetically prepared by the catalysis of *C*-glycosyltransferases (CGTs). However, while a large family of *O*-glycosyltransferases (OGTs) is known, a limited number of CGTs have been discovered in plants [88]. Many detailed reviews on the chemical synthesis of aryl-*C*-glycoside have been published [8,89,90,91,92,93]. Herein, a brief historical review of the total synthesis of natural aryl-*C*-glycosides is presented. From a retrosynthetic viewpoint, the strategy of synthesis of aryl-*C*-glycoside usually includes two protocols, as described below.

### 4.1. C-Glycosylation of Arenes and De Novo Construction of the Aromatic Moiety

**Scheme 1:**
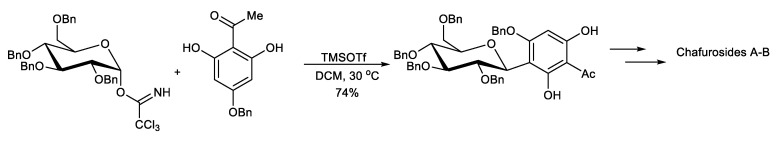
Concise synthesis of chafurosides A and B.

**Scheme 2:**
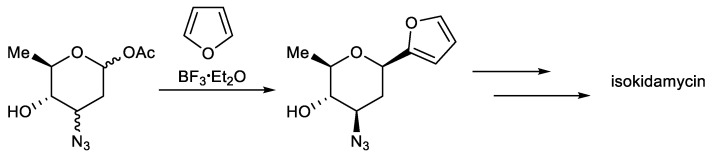
Total synthesis of isokidamycin.

**Scheme 3:**
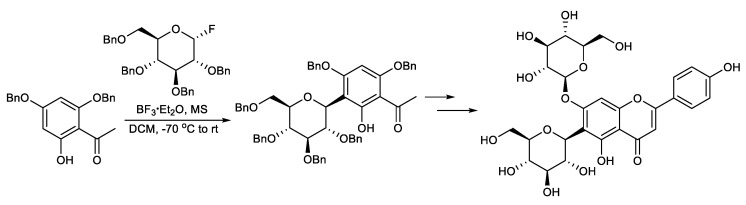
Total synthesis of saponarin.

**Scheme 4:**
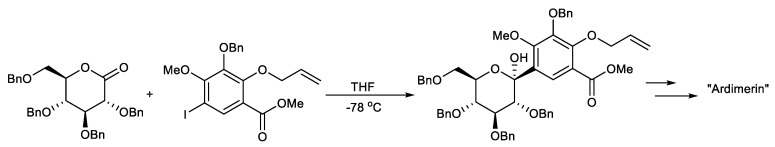
Total synthesis of the proposed structure of ardimerin.

**Scheme 5:**
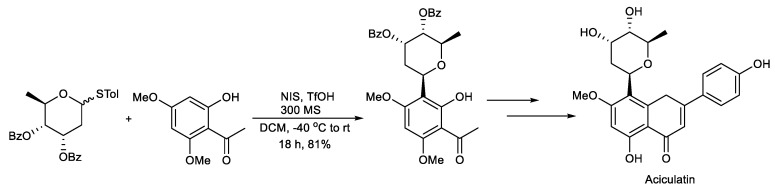
Synthesis of aciculatin.

This protocol exploits the installation of a sugar moiety on a full or partial aromatic structure. For a partial aryl-*C*-glycoside structure, it uses a simple *C*-glycoside as the starting material, which is functionalized, providing a complex aryl unit. The classical electrophilic aromatic substitution approach was often applied. The sugar portion includes glycosyl trichloroacetimidate (Scheme 1) [94], glycosyl acetate (Scheme 2) [95], glycosyl fluoride (Scheme 3) [96], glycosyl lactone (Scheme 4) [97], glycosyl thioglycoside (Scheme 5) [98], and other sugars. This strategy was successfully applied to the total synthesis of pluramycins [99], aciculatin [98], vineomycin B_2_ and its methyl ester [100,101,102], angucycline C5 glycosides [103], paecilomycin B [104,105], aspalathin [106], nothofagin [106], chrysomycin A [107], vicenin-2 [108], 3,3′-Di-*O*-methyl Ardimerin [109], deacetylravidomycin M [110], aquayamycin [111], a precursor of kendomycin [112], and anthraquinone-based aryl-*C*-glycosides [113].

Toshiyuki Kan et al. reported the regioselective synthesis of chafurosides A (**107**) and B (**108**) using a novel protecting-group strategy. The construction of the dihydrofuran ring was achieved via an intramolecular Mitsunobu reaction. The key step in the *C*-glycosylation is the O→C rearrangement of the phenolic glycoside formed by TMSOTf-catalyzed glycosylation (Scheme 1) [94].

Martin et al. reported the total synthesis of isokidamycin, which features the use of a silicon tether as a disposable regiocontrol element in an intramolecular Diels–Alder reaction between a substituted naphthyne and a glycosyl furan and a subsequent *O*→*C*-glycoside rearrangement (Scheme 2) [95].

Sato et al. reported the total synthesis of saponarin. Saponarin was efficiently synthesized via 11 steps from 2, 4-*O*-dibenzylphloroacetophenone, with an overall yield of 37%. The key step also features a glycosylation involving per-*O*-benzylglucosyl α-fluoride and 2, 4-*O*-dibenzylphloroacetophenone, applying the *O*→*C* glycoside rearrangement method (Scheme 3) [96].

Suzuki K. et al. reported the total synthesis of the proposed structure of ardimerin, whose key step includes the *β*-selective formation of the crucial *C*-glycoside linkage by the reaction between aryl iodide, through a halogen–metal exchange reaction, and lactone (Scheme 4) [97].

Lee et al. reported the total synthesis of aciculatin. The key step is the glycosylation of the digitoxosyl thioglycoside with an electron-rich phenol activated by NIS/TfOH, which afforded the *β*-D-digitoxopyranoside (Scheme 5) [98].

Besides the nucleophilic attack reaction of the aryl portions to provide aryl-*C*-glycoside molecules, the transition metal-catalyzed cross-coupling reactions to form the C_sp3_-C_sp2_ bond of aryl-*C*-glycosides are becoming more and more powerful. The Negeshi [114], Kumada [115], Stille [116,117,118], Heck [119,120], Sonogashira [121], Hiyama [122], and radical [123,124,125] cross-coupling reactions with different metals such as Pd [126,127], Ni [128,129], Fe [124,125], Co [115], Ir [130] as catalysts, as well as C-H activation [131,132] were successfully applied to the construction of aryl-*C*-glycoside scaffolds.

### 4.2. De Novo Construction of the Sugar Moiety

This approach uses the reactions of assembled aryl units based on methods for the construction of a sugar, including, but not limited to (a) the hetero-Diels–Alder reaction, (b) the 1,3-dipolar cycloaddition of nitrile oxides, (c) the ring-closing olefin metathesis [8], the de novo asymmetric approach [133,134,135], and the ring opening-ring closure strategy [136]. It was successfully applied to the synthesis of *O*-spiro-*C*-aryl glycosides [137] and 2-deoxy-*β*-*C*-aryl glycosides [138].

**Scheme 6:**
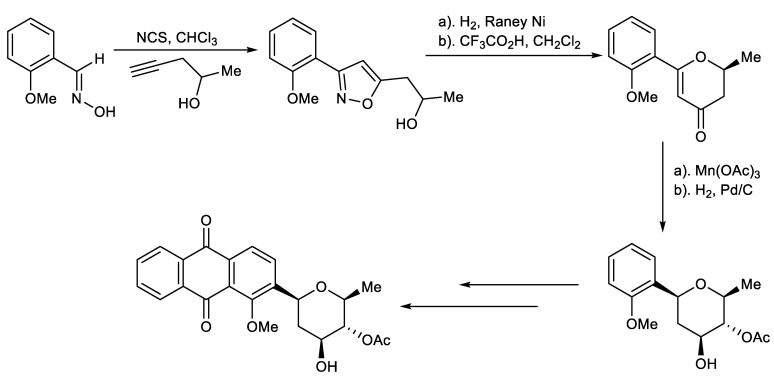
Total synthesis of naturally occurring *C*-aryl glycosides.

Hauser et al. reported that de novo synthesis of *C*-aryl glycosides based on cycloaddition of an aryl nitrile oxide with 4-pentyn-2-ol, which was straightforwardly converted to the pyranone through sequential hydrogenolysis of the N-O bond of the isoxazole followed by acid-catalyzed intramolecular cyclization. (Scheme 6) [139].

**Scheme 7:**
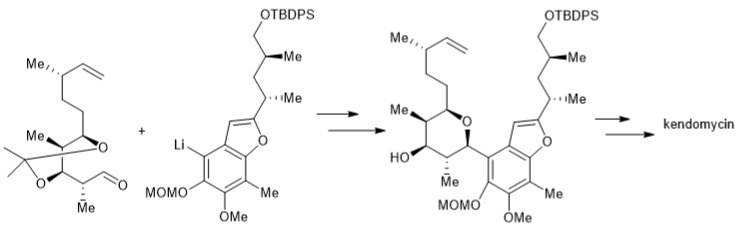
Concise synthesis of kendomycin.

Johann Mulzer et al. reported the development of a convergent and concise route to an advanced precursor of kendomycin by applying an S_N_1 ring cyclization as a key step. The sugar moiety formed by the acid-catalyzed intramolecular etherification reaction of aryl-substituted 1, 3, 5-triol (Scheme 7) [140].

## 5. Conclusions and Perspectives

In this review, the recently discovered aryl-*C*-glycoside structures were listed and their biological activities as well as their synthetical approaches were summarized. The diverse structures of natural aryl-*C*-glycosides and their multiple pharmacological effects suggest significant medicinal applications. This review presents a summary of studies published from 2002 to date on this promising compounds. The core structure of aryl-*C*-glycosides, which is glycosylated and/or acylated, exhibits a great number of molecular entities and possesses various pharmaceutical effects. It is expected that more and more aryl-*C*-glycoside scaffolds including natural and synthetical molecules will be disclosed, and their medicinal application will come true in the near future to benefit human health.

## Figures and Tables

**Table 1:** Structures, Bioactivities, Sources of Aryl-*C*-glycoside Molecules.

Entry	Name	Carbohydrate	Bioactivities	Source	Ref.
1	Apigenosylide B (**16**)	*β*-D-glucose	α-glucosidase inhibitory activity	*Machilus japonica*	[9]
2	Speciflavoside A (**32**)	*β*-D-glucose	antiviral activity	*Lilium speciosum* var. *gloriosoides Baker*	[10]
3	Compounds **34**–**36**	*β*-D-glucose	cytotoxicity activity	*Lemna japonica*	[11]
4	Nelumboside B (**40**)	*β*-D-glucose	antioxidant activity	*Nelumbo nucifera*	[12]
5	Compound **42**	*β*-D-glucose	antioxidant activity	*Gentiana piasezkii*	[13]
6	Compounds **45**–**46**	*β*-D-glucose	anti-complementary activity	*Trollius chinensis*	[14]
7	Compound **68**	*β*-D-glucose	macrophage respiratory burst inhibitory activity	*Cyperus rotundus*	[15]
8	Diandraflavone (**81**)	*β*-D-glucose	selective inhibition on superoxide anion	*Drymaria diandra*	[16]
9	Nervilifordin J (**94**)	*β*-D-galactose	anti-inflammatory activity	*Nervilia fordii*	[17]
10	Chafurosides A–B (**107**–**108**)	*β*-D-mannose	anti-inflammation	oolong tea	[18]
11	Compound **109**	*β*-L-fucose	glycation inhibitory activity	the style of *Zea mays* L.	[19]
12	Compound **112**	*β*-L-boivinose	glycation inhibitory activity	the Style of *Zea mays*	[20]
13	Glomexanthones A–C (**131**, **129**, **132**)	*β*-D-glucose	neuroprotective effects	*Polygala glomerata*	[21]
14	Shamimoside (**135**)	*β*-D-glucose	antioxidant activity	*Bombax ceiba*	[22]
15	Compound **136**	*β*-D-glucose	neuroprotective activity	*Swertia punicea*	[23]
16	Calophymembranside C (**141**)	*β*-D-glucose	transcriptional inhibitory activity of RXRα	*Calophyllum membranaceum*	[24]
17	Arenicolin A (**152**)	*β*-D-glucose	anticancer activity	*Penicillium arenicola*	[25]
18	Compound **155**	*β*-D-glucose	antipyretic activity	*Melicope pteleifolia*	[26]
19	Ardimerin digallate (**169**)	*β*-D-glucose	inhibitory activity on HIV-1 and HIV-2 ribonuclease H	*Ardisia japonica*	[27]
20	Kunzeachromones A–F (**172**–**177**)	*β*-D-glucose	antivirus activity	*Kunzea ambigua.*	[28]
21	Neopetrosins A–D (**180**–**183**)	*α*-D-mannose	hepatoprotective activity	*Neopetrosia chaliniformis*	[29]
22	Konamycins A–B (**188**–**189**)	*β*-D-amicetose	radical scavenging activity	*Streptomyces hyaluromycini* MB-PO13^T^	[30]
23	Monacyclinones I, J (**195**, **192**)	*α*-L-amino sugar	anticancer activity	*Streptomyces* sp. HDN15129	[31]
24	Marmycins A and B (**198**–**199**)	*α*-L-amino sugar	anticancer activity	actinomycete related to the genus *Streptomyces*	[32]
25	Aciculatin (**200**)	*β*-D-digitoxopyranose	cytotoxic, anti-inflammatory, anti-arthritis activity	*Chrysopogon aciculatus*	[33]
26	Compound **201**	*β*-D-digitoxopyranose	anticancer activity	*Chrysopogon aciculatis*	[33]
27	Compound **202**	*β*-D-boivinopyranose	anticancer activity	*Chrysopogon aciculatis*	[33]
28	Aciculatinone (**204**)	3-keto-*β*-D-digitoxopyranose	anticancer activity	*Chrysopogon aciculatis*	[33]
29	Isocassiaoccidentalin B (**206**)	*β*-D-6-Deoxy-ribo-hexos-3-ulose	free-radical scavenging activity	*Cassia nomame*	[34]
30	Grincamycins B–D (**207**–**209**)	*β*-D-olivose	anticancer activity	*Streptomyces lusitanus* SCSIO LR32	[35]
31	Marangucyclines A–B (**210**–**211**)	*β*-D-olivose	anticancer activity	*Streptomyces* sp. SCSIO 11594	[36]
